# Supplementary material for: A central role of IKK2 and TPL2 in JNK activation and viral B-cell transformation
Source: Nat Commun. 2020 Feb 4;11:685. doi: 10.1038/s41467-020-14502-x (PMC7000802; doi:10.1038/s41467-020-14502-x)
Supplement: Supplementary file 1 — Supplementary Information [file 41467_2020_14502_MOESM1_ESM.pdf]

A central role of IKK2 and TPL2 in JNK activation and viral B-cell transformation

Stefanie Voigt, Kai R. Sterz, Fabian Giehler, Anne-Wiebe Mohr,
Joanna B. Wilson, Andreas Moosmann, Arnd Kieser

Supplementary Figures, Tables and References

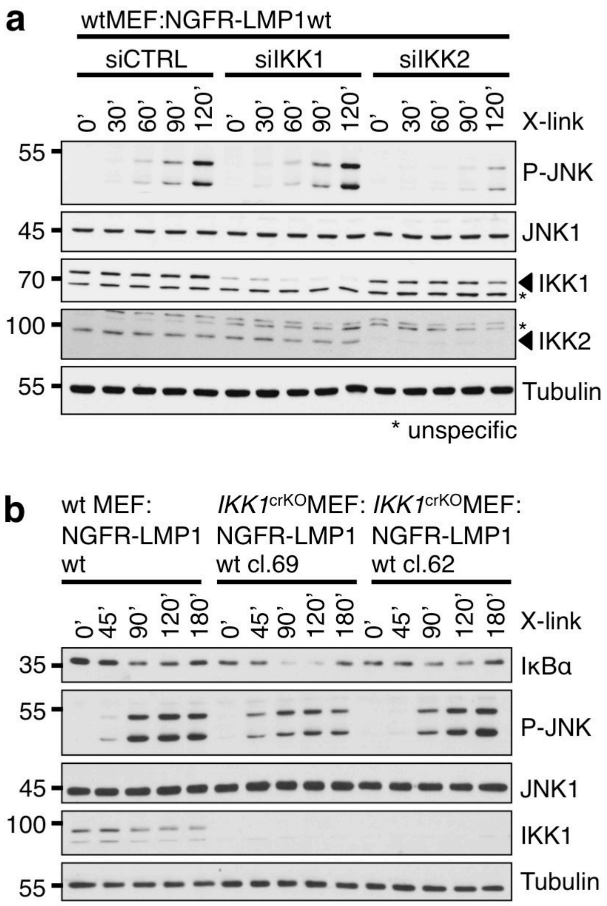

Supplementary Figure 1

Supporting evidence for the differential requirement of IKK1 and IKK2 in JNK activation by LMP1. a The siRNA-mediated knockdown of IKK2, but not of IKK1, blocks JNK1 activation by LMP1. Wildtype MEF:NGFR-LMP1wt cells were transfected twice with siRNA targeting IKK1 (siIKK1), IKK2 (siIKK2), or non-targeting control siRNA (siCTRL) in six-well-plates. One day after the last transfection, NGFR-LMP1 was activated by antibody crosslinking and JNK activation was monitored. The knockdown of IKK1 or IKK2 was confirmed by immunoblotting using the indicated antibodies. **b** No defects in JNK activation by LMP1 in an additional CRISPR/Cas9 IKK1 knockout clone. *IKK1*^{crKO}MEF:NGFR-LMP1wt clones 62 and 69 were induced by antibody crosslinking and analysed by immunoblotting as indicated. The knockout of IKK1 was verified at the protein level by the IKK1 antibody and by sequencing (see Supplementary Table 2).

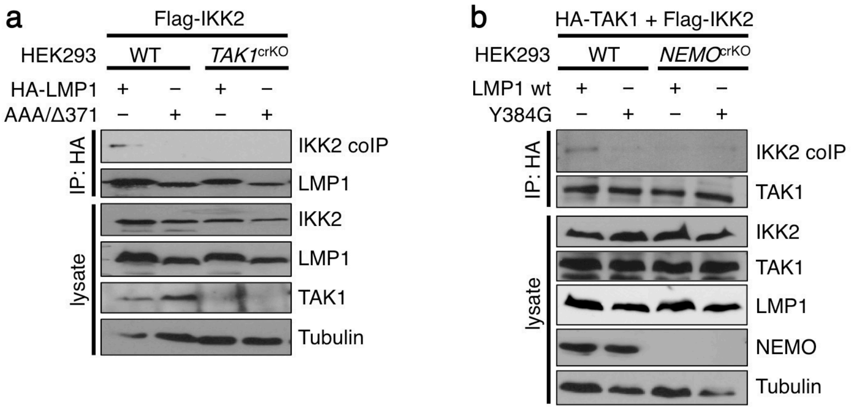

Supplementary Figure 2

TAK1 and NEMO mediate signaling complex formation and IKK2 recruitment at LMP1-CTAR2 a The knockout of TAK1 prevents LMP1 interaction with IKK2. The experiment was performed as described in Figure 3f, except that co-immunoprecipitation of LMP1 and IKK2 was performed vice versa. HA-LMP1 was immunoprecipitated via the HA (3F10) antibody and co-precipitating Flag-IKK2 was detected by the IKK2 antibody. **b** NEMO is required for the CTAR2-induced interaction of TAK1 with IKK2. NEMO was targeted by CRISPR/Cas9 in HEK293 cells. HEK293 wildtype and *NEMO*^{crKO} (clone 28) cells were transfected with Flag-IKK2 and HA-TAK1 together with pSV-LMP1 wildtype or pSV-LMP1(Y384G). HA-TAK1 was immunoprecipitated via the HA (3F10) antibody and interacting Flag-IKK2 was detected by the H-470 antibody.

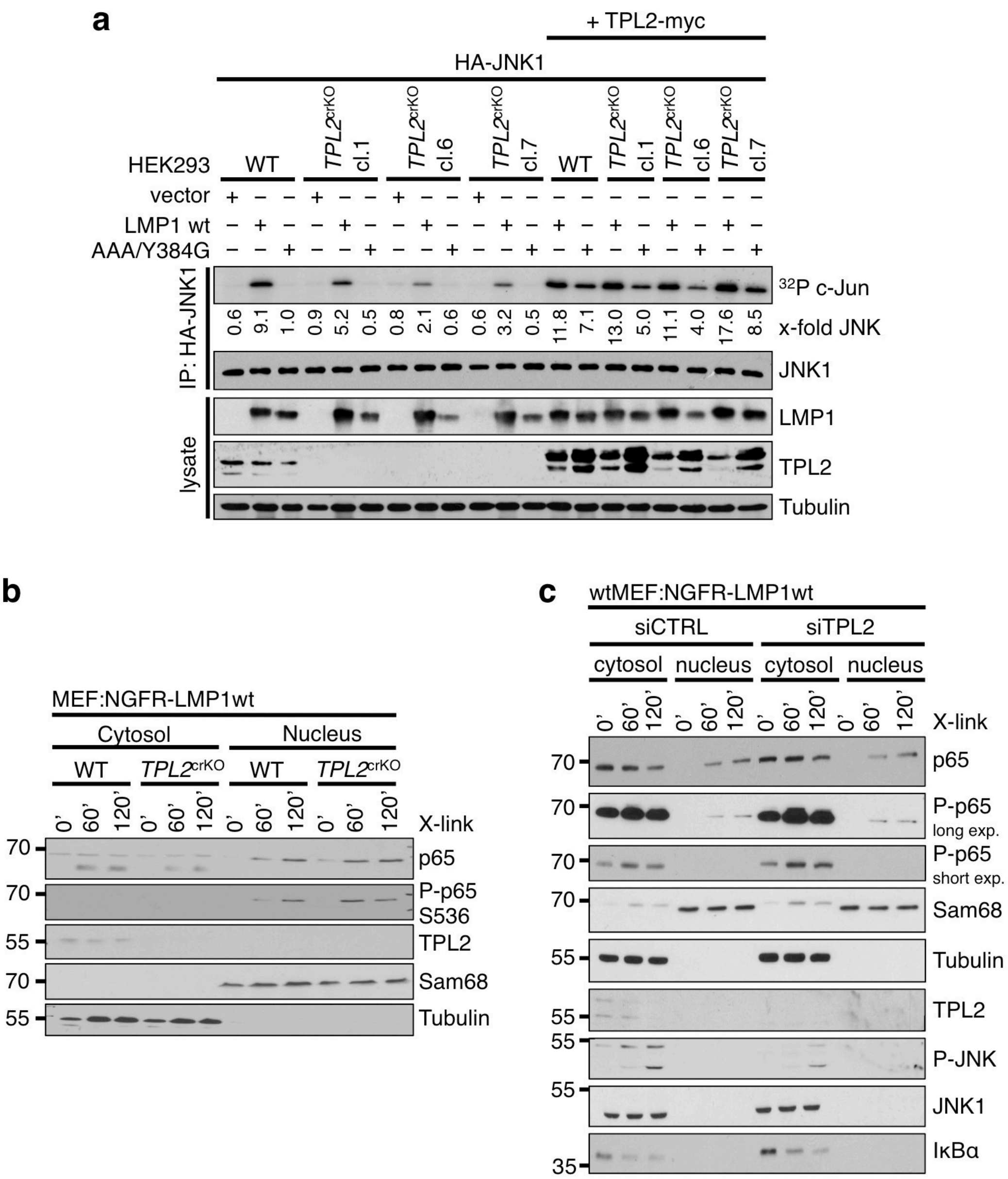

Supplementary Figure 3

Supportive data for the differential role of TPL2 in JNK and NF-κB activation by LMP1. a The knockout of TPL2 in HEK293 cells interferes with LMP1's ability to induce the JNK pathway and exogenous TPL2 expression rescues JNK activation. The *TPL2* gene was targeted in HEK293 cells by CRISPR/Cas9. Wildtype HEK293 cells and the TPL2 knockout clones 1, 6 and 7 were transfected with HA-JNK1 together with LMP1 wildtype, the inactive AAA/Y384G mutant, or empty vector. Where indicated, TPL2-myc was co-transfected. HA-JNK1 was immunoprecipitated and its activity was monitored in *in vitro* kinase assays using GST-c-Jun as substrate. Quantification of JNK activity is given as x-fold induction versus wildtype cells transfected with AAA/Y384G, normalised to the amounts of immunoprecipitated HA-JNK. Ectopic expression of kinase-active TPL2-myc itself caused a certain activation of JNK. However, TPL2-myc facilitated high levels of JNK activation by LMP1 in TPL2-deficient cells. *TPL2* gene targeting was verified by

immunoblotting and sequencing (see Supplementary Table 2). **b** The knockout of TPL2 has no negative effect on LMP1-induced p65 NF-κB translocation into the nucleus or p65 NF-κB phosphorylation at serine 536. NGFR-LMP1 was induced in wildtype and *TPL2*^{crKO} (clone 8) cells for the indicated times. Cytoplasmic and nuclear fractions were prepared and analysed by immunoblotting. **c** The knockdown of TPL2 does not affect LMP1-induced p65 NF-κB phosphorylation at serine 536 or its translocation into the nucleus. Wildtype MEF:NGFR-LMP1wt cells were transfected twice with siRNA targeting murine TPL2 (siTPL2) or non-targeting siRNA (siCTRL). NGFR-LMP1 was induced by antibody crosslinking for the indicated times. Cytoplasmic and nuclear fractions were prepared and further analysed by immunoblotting.

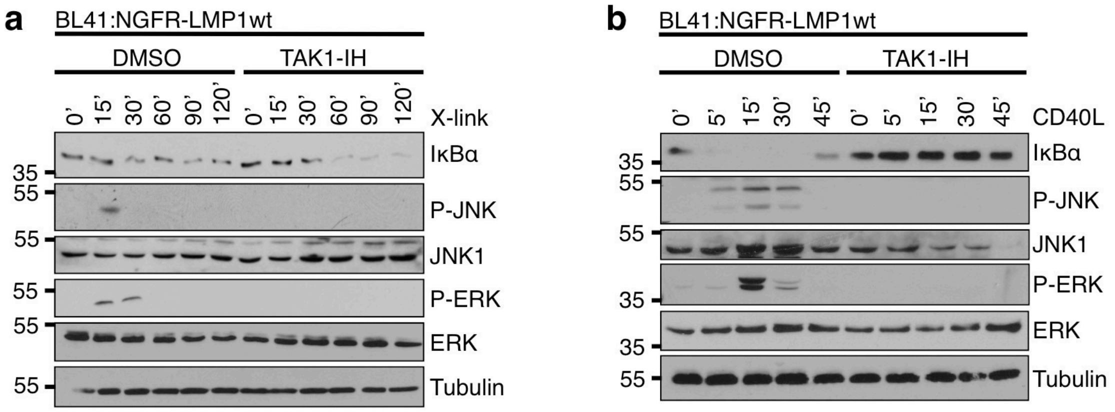

Supplementary Figure 4

LMP1 and CD40 signaling in B-cells differ with regard to the functions of TAK1. **a, b** BL41:NGFR-LMP1wt cells were treated with solvent (DMSO) or 500 nM (5Z)-7-oxozeaenol (TAK1-IH). In (**a**) NGFR-LMP1 activity was induced by antibody crosslinking (X-link), in (**b**) CD40 was activated with 600 ng/ml soluble CD40 ligand (CD40L) for the indicated times. JNK, ERK and NF-κB activation was detected in total cell lysates using the indicated antibodies.

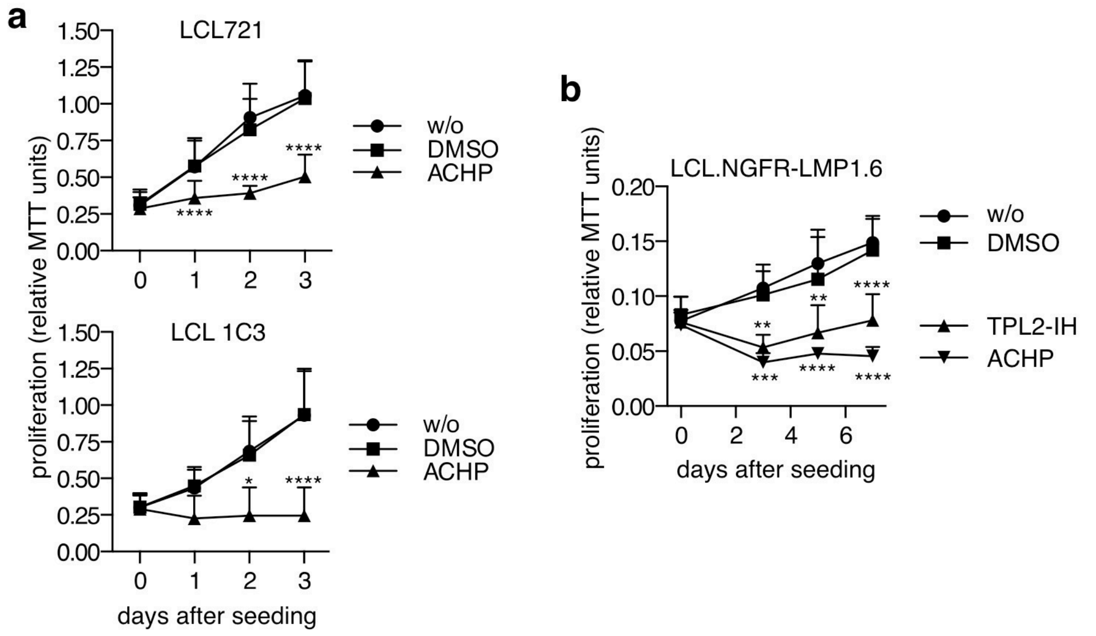

Supplementary Figure 5

Effects of pharmacological IKK and TPL2 inhibition on lymphoblastoid cells. a IKK inhibition interferes with proliferation of LCLs. LCL721 and LCL 1C3 lymphoblastoid cell lines were either kept in culture without further treatment, in the presence of solvent (DMSO) or 10 µM ACHP. Cell proliferation was measured at the indicated times as MTT conversion. Data are mean values ±SD of 5 biological replicates. **b** IKK and TPL2 inhibition blocks proliferation of LCLs driven by NGFR-LMP1. LCL.NGFR-LMP1.6 cells were kept under permanent antibody crosslink to activate NGFR-LMP1 signaling (w/o). As indicated, the cells were additionally incubated with DMSO, 10 µM ACHP, or 10 µM TC-S7006 (TPL2-IH). MTT assays were performed to monitor proliferation. Data are mean values ±SD of 5 biological replicates. **a, b** Statistical analysis was performed with two-way ANOVA at alpha 0.05, p-values: *p≤0.05, **p≤0.01, ***p≤0.001, ****p≤0.0001.

```
                                  PxQxT  212
PTLD880      187 HGQRHSDEHHHDDSLPHPQQATDDSAHESDSNSNEGRHHLLVSGAGDGPPLCSQNLGAPG 246
PTLD099      187 HGQRHSDEHHHDDSLPHPQQATHNSSHESDSNSNEGRHHLLVSGAGDGPPLCSQNLGSPG 246
LMP1 B95.8   187 HGQRHSDEHHHDDSLPHPQQATDDSGHESDSNSNEGRHHLLVSGAGDGPPLCSQNLGAPG 246
                 **********************  * *******************************.**

PTLD880      247 GGPDNGPQDPDNTDDNGPQDPDNTDDNGPHDPLPQDPDNTDDNGPQDPLPQDPDNTDDNG 306
PTLD099      247 GGPDNGPQDPDNTDDNGPQDPDNTDDNGPHDPL--------------------------- 279
LMP1 B95.8   247 GGPDNGPQDPDNTDDNGPQDPDNTDDNGPHDPL--------------------------- 279
                 *********************************

PTLD880      307 PQDPLPQDPDNTDDNGPHDPLPQDPDNTDDNGPQDPDNTDDNGPHDPLPHSPSDSAGNDG 366
PTLD099      280 ---------------------PQDPDNTDDNGPQDPDNTDDNGPHDPLPHSPSDSAGNDG 318
LMP1 B95.8   280 ---------------------PQDPDNTDDNGPQDPDNTDDNGPHDPLPHSPSDSAGNDG 318
                                      ***************************************
                         328                                   366
PTLD880      367 GPPQLTEEVQNKGGDQGPPLMTDGGGGHSHDSGHRGGDPHLPTLLLGTSGSGGDDDDPHG 426
PTLD099      319 GPPQLTEEVQNKGGDQGPPLMTDGGGGHSHDSGHGGGDPHLPTLLLGTSGSGGDDDDPHG 378
LMP1 B95.8   319 GPPQLTEEVENKGGDQGPPLMTDGGGGHSHDSGHGGGDPHLPTLLLGSSGSGGDDDDPHG 378
                 *********.************************ ************.************

PTLD880      427 PVQLSYYD* 434
PTLD099      379 PVQLSYYD* 386
LMP1 B95.8   379 PVQLSYYD* 386
                 *********
```

Supplementary Figure 6

Signaling domains of LMP1 proteins expressed in PTLD099 and PTLD880 cells established from post-transplantation lymphoproliferative disease tumour biopsies. EBV-positive B-cells were isolated from two independent PTLD tumour biopsies derived from two PTLD patients. Genomic DNA was isolated from PTLD099 and PTLD880 cells, the *LMP1* genes were amplified by PCR and the LMP1 signaling domains of both samples were sequenced. Primer sequences are given in Supplementary Table 1. Both tumour-derived LMP1 versions carry mutations as compared to proto-type B95.8 LMP1, which have previously been observed in *LMP1* genes of field isolates [1-3]. Most stinkingly, PTLD880 LMP1 carries an insertion of 33 bp repeats after Leu 279 comprising 48 amino acids. The mutational hotspots Gly 212 (destruction box), Glu 328 (JAK3 box 2) and Ser 366 are altered in both tumour-derived LMP1 proteins. Interestingly, PTLD099 carries an unusual mutation of two amino acids proximal to the TRAF interaction site PxQxT of CTAR1. Aspartates 209 and 210 are replaced in PTLD099 LMP1 by histidine and asparagine. Numbering of LMP1 residues refers to amino acid positions in B95.8 LMP1. The PxQxT motif is framed. Mutations in PTLD LMP1 proteins versus B95.8 LMP1 are highlighted in red.

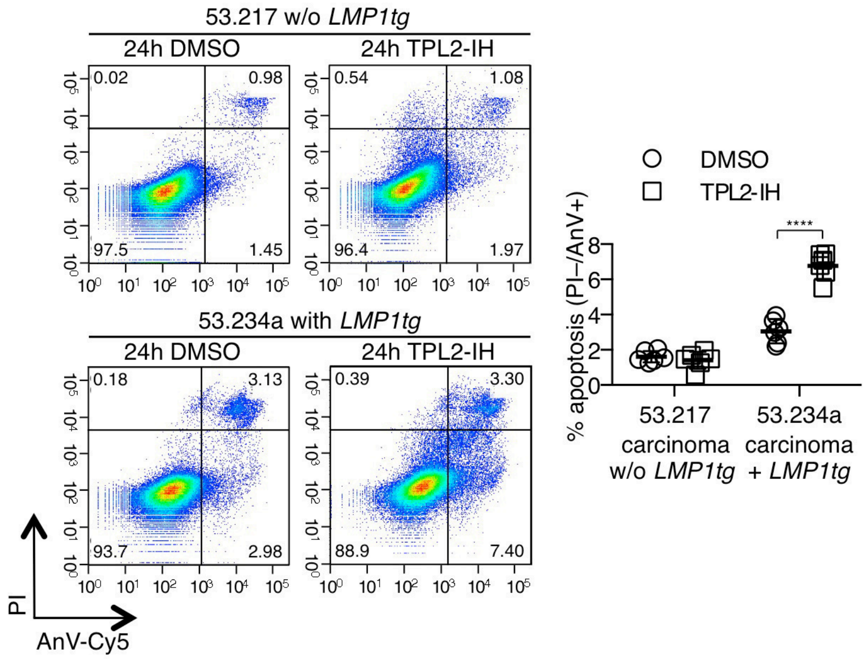

Supplementary Figure 7

LMP1-dependent carcinoma cells derived from LMP1-transgenic mice show a significant increase in apoptosis after TPL2 inhibition. The LMP1-negative murine carcinoma line 53.217 and the corresponding LMP1-dependent carcinoma line 53.234a, which was established from *LMP1*-transgenic (*LMP1tg*) mice, were treated with 10 µM TC-S7006 (TPL2-IH) or DMSO for 24 h. Cells were stained with propidium iodide (PI) and Annexin V-Cy5, and further analysed by flow cytometry. PI-/AnV-Cy5+ cell numbers indicate apoptosis rates. Combined apoptosis rates of six independent experiments are given as mean values ± SD. Statistical analysis was performed with two-way ANOVA at alpha 0.05, p-value: ****$p \leq 0.0001$.

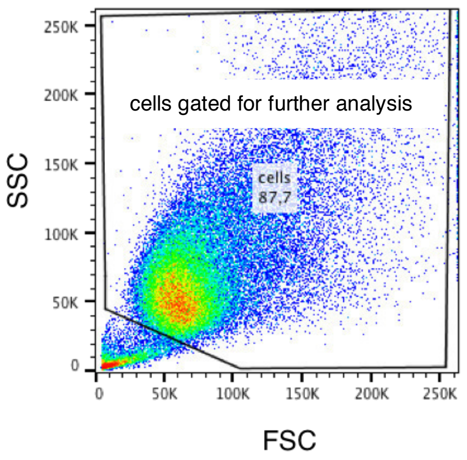

Supplementary Figure 8
Gating strategy. Lymphocytes and carcinoma cells within the indicated gate were further analysed for PI and AnV-Cy5 staining. By this gating strategy all cells were included into the analysis whereas cell debris was excluded.

Supplementary Table 1
PCR and sequencing primers used in this study.

Name	Sequence	Application
AK549	CACCACTATGCCTGGCTTGA	murine *NEMO* ex3 forward primer
AK550	TCTGGATGGTTCTTGGGCAC	murine *NEMO* ex3 reverse primer
AK551	TGCTGTGAATGGAAGTCTAG	murine *NEMO* ex3 sequencing primer
AK552	AGGCCCTTTGGTCTCCAATG	murine *TPL2* ex2 forward primer
AK553	TGGCAGCCATTCTTAAGCCA	murine *TPL2* ex2 reverse primer
AK554	CAAACACATTATAGTGAAGG	murine *TPL2* ex2 sequencing primer
SV008	ATAGGAACCGCACGCACGAT	murine *IKK1* ex1 forward primer
SV009	TCTAGTCCAGTCTCCAGTGG	murine *IKK1* ex1 reverse primer
SV010	GACTCTGCATGCGGAAGCTA	murine *IKK1* ex1 sequencing primer
AK555	TCCCATGGGTCTTGAATGCA	human *TPL2* ex2 forward primer
AK556	CATGTTGGCCAGACTGGTCT	human *TPL2* ex2 reverse primer
AK557	TGCAAGTGAAGAGCCAGCAG	human *TPL2* ex2 sequencing primer
AK558	AGCACCTCCCTTTGGAATGG	human *NEMO* ex2 forward primer
AK559	ACAGAGGCCTGGACATGTTG	human *NEMO* ex2 reverse primer
AK560	GCATCCTGATACACTAGGTG	human *NEMO* ex2 sequencing primer
AK575	TGCTTCTTAGCTTACTCCAC	human *TAK1* ex1 forward primer
AK576	CACAGCCCTCAGTAACGTGG	human *TAK1* ex1 reverse primer
AK577	AAAACGCAGCGGAGCCAGAG	human *TAK1* ex1 sequencing primer
AK605	CAACTGCCTTGCTCCTGACACAC	*LMP1* forward primer
AK606	GGGAGTGTGTGCCAGTTAAGGTG	*LMP1* reverse primer
AK607	GTGATTAGCTAAGGCATTCC	*LMP1* sequencing primer

Supplementary Table 2
Sequencing results of CRISPR/Cas9-mediated gene knockout cell clones.

Cell line[a] and gene	Accession number	Sigma-Aldrich gRNA ID	Ex[b]	Mutations at gRNA targeting site
MEF *IKK1*^{crKO} cl. 62	NM_007700.2	MM0000145296	1	Indel, frameshift
MEF *IKK1*^{crKO} cl. 69	NM_007700.2	MM0000145296	1	Indel, frameshift
MEF IKK2 $^{-/-}$ *IKK1*^{crKO} cl.8	NM_007700.2	MM0000145296	1	Indel, frameshift
MEF *NEMO*^{crKO} cl.2	NC_000086.7	MM0000125118	3	Indel, frameshift
MEF *NEMO*^{crKO} cl.7	NC_000086.7	MM0000125118	3	Indel, frameshift
MEF *NEMO*^{crKO} cl.19	NC_000086.7	MM0000125118	3	Indel, frameshift
MEF *TPL2*^{crKO} cl.8	NC_000084.6	MM0000226028	2	Indel, frameshift
HEK293 *Tpl2*^{crKO} cl.1	NC_000010.11	HS0000206639	2	Indel, frameshift
HEK293 *TPL2*^{crKO} cl.6	NC_000010.11	HS0000206639	2	Indel, frameshift
HEK293 *TPL2*^{crKO} cl.7	NC_000010.11	HS0000206639	2	Indel, frameshift
HEK293 *TAK1*^{crKO} cl.3	NG_011966.2	HS00000272768	1	Indel, frameshift
HEK293 *NEMO*^{crKO} cl.28	NG_009896.1	HS0000099337	2	Indel, translational start site deleted

[a] All mouse embryonic fibroblast lines express NGFR-LMP1
[b] Exon targeted by gRNA

Voigt et al.

Supplementary Table 3. Quantification and statistics of immunoblots (Figures 1 to 6)

n, number of biological replicates
SD, standard deviation

Figure	Antibody	Sample	X-link	Mean (n=3)	±SD	p-value ($Y_{384}G$ vs. corresponding wt)
**Figure 1c**	P-JNK	NGFR-LMP1wt	0 min	0.03	0.02	
			20 min	0.05	0.05	
			30 min	0.06	0.07	
			45 min	0.26	0.15	
			60 min	0.54	0.20	
			90 min	0.89	0.14	
			§120 min	1.00	0.00	
			180 min	0.91	0.03	
			360 min	0.84	0.31	
			600 min	0.42	0.14	
		NGFR-LMP1($Y_{384}G$)	0 min	0.03	0.04	
			20 min	0.04	0.04	p = 0.80
			30 min	0.03	0.04	p = 0.58
			45 min	0.04	0.04	p = 0.067
			60 min	0.04	0.05	*p = 0.014
			90 min	0.04	0.05	***p = 0.00059
			120 min	0.06	0.07	****p = 0.000019
			180 min	0.05	0.06	****p = 0.000018
			360 min	0.12	0.11	*p = 0.018
			600 min	0.04	0.04	*p = 0.011

§set to 1 T-test, unpaired, two-tailed

	Antibody	Sample	X-link	Mean (n=3)	±SD	p-value ($Y_{384}G$ vs. corresponding wt)
	IκBα	NGFR-LMP1wt	§0 min	1.00	0.00	
			20 min	0.75	0.12	
			30 min	0.63	0.18	
			45 min	0.39	0.29	
			60 min	0.28	0.14	
			90 min	0.28	0.19	
			120 min	0.31	0.17	
			180 min	0.31	0.16	
			360 min	0.29	0.26	
			600 min	0.48	0.17	
		NGFR-LMP1($Y_{384}G$)	§0 min	1.00	0.00	
			20 min	1.21	0.13	**p = 0.01
			30 min	1.13	0.08	*p = 0.011
			45 min	1.10	0.09	*p = 0.016
			60 min	0.94	0.15	**p = 0.0049
			90 min	0.83	0.20	*p = 0.026
			120 min	1.11	0.38	*p = 0.029
			180 min	1.14	0.38	*p = 0.025
			360 min	1.07	0.43	p = 0.054
			600 min	0.87	0.46	p = 0.24

§set to 1 T-test, unpaired, two-tailed

	Antibody	Sample	X-link	Mean (n=3)	±SD	p-value ($Y_{384}G$ vs. corresponding wt)
**Figure 1d**	TRAF3	NGFR-LMP1wt	§0 min	1.00	0.00	
			60 min	0.40	0.07	
			120 min	0.43	0.00	
		NGFR-LMP1($Y_{384}G$)	§0 min	1.00	0.00	
			60 min	0.37	0.20	p = 0.82
			120 min	0.40	0.29	p = 0.86

§set to 1 T-test, unpaired, two-tailed

Figure 1e	Antibody	Sample	X-link	Mean (n=6)	±SD	p-value (*IKK2* -/- vs. corresponding WT)
	IκBα	WT	[§]0 min	1.00	0.00	
			30 min	0.87	0.15	
			60 min	0.46	0.23	
			90 min	0.40	0.26	
			120 min	0.46	0.26	
			180 min	0.49	0.15	
		IKK2 -/-	[§]0 min	1.00	0.00	
			30 min	0.94	0.13	p = 0.43
			60 min	1.09	0.49	*p = 0.016
			90 min	0.97	0.32	**p = 0.008
			120 min	1.05	0.41	*p = 0.015
			180 min	1.11	0.79	p = 0.085

[§]set to 1

T-test, unpaired, two-tailed

	Antibody	Sample	X-link	Mean (n=2)	±SD	p-value (*IKK2* -/- nucleus vs. corresponding WT nucleus)
**Figure 1f**	p65	WT, cytoplasm	[§]0 h	1.00	0.00	
			1 h	0.55	0.26	
			2 h	0.50	0.24	
		IKK2 -/-, cytoplasm	[#]0 h	1.00	0.00	
			1 h	0.87	0.01	
			2 h	0.84	0.18	
		WT, nucleus	0 h	0.00	0.00	
			1 h	0.84	0.46	
			2 h	0.80	0.09	
		IKK2 -/-, nucleus	0 h	0.00	0.00	
			1 h	0.01	0.01	p = 0.12
			2 h	0.00	0.00	**p = 0.0056

[§]set to 1 for WT
[#]set to 1 for *IKK2* -/-

T-test, unpaired, two-tailed

	Antibody	Sample	X-link	Mean (n=6[†])	±SD	p-value (*IKK2* -/- vs. corresponding WT)
**Figure 2a**	P-JNK	WT	0 min	0.03	0.04	
			30 min	0.12	0.13	
			60 min	0.42	0.23	
			90 min	0.79	0.28	
			[§]120 min	1.00	0.00	
			180 min	1.22	0.51	
		IKK2 -/-	0 min	0.01	0.01	
			30 min	0.01	0.02	p = 0.062
			60 min	0.02	0.03	**p = 0.0018
			90 min	0.07	0.10	***p = 0.00013
			120 min	0.09	0.13	****p = 0.000000013
			180 min	0.05	0.06	***p = 0.00024

[§]set to 1

T-test, unpaired, two-tailed

[†]stimulation times were included into the quantitative analysis, which were tested in all biological replicates

	Antibody	Sample	X-link	Mean (n=3)	±SD	p-value (ACHP vs. corresponding DMSO)
**Figure 2b**	P-JNK	DMSO	0 min	0.01	0.01	
			30 min	0.02	0.01	
			60 min	0.19	0.17	
			90 min	0.71	0.38	
			[§]120 min	1.00	0.00	
			180 min	0.87	0.14	
		ACHP	0 min	0.00	0.00	
			30 min	0.00	0.00	
			60 min	0.01	0.01	p = 0.14
			90 min	0.04	0.02	*p = 0.039
			120 min	0.09	0.06	****p = 0.000013
			180 min	0.12	0.13	**p = 0.0025

[§]set to 1

T-test, unpaired, two-tailed

	Antibody	Sample	TNFα	Mean (n=3[†])	±SD	p-value (*IKK2* -/- vs. corresponding WT)
**Figure 2c**	P-JNK	*IKK2* -/-	0 min	0.03	0.04	
			10 min	0.46	0.15	**p = 0.0029
			20 min	0.64	0.19	p = 0.41
			30 min	0.13	0.09	p = 0.73
		WT	0 min	0.05	0.07	
			[§]10 min	1.00	0.00	
			20 min	0.49	0.20	
			30 min	0.10	0.11	

[§]set to 1

T-test, unpaired, two-tailed

[†]stimulation times were included into the quantitative analysis, which were tested in all biological replicates

Antibody	Sample	TNFα	Mean (n=3*)	±SD	p-value (*IKK2* -/- vs. corresponding WT)
IκBα	*IKK2* -/-	[§]0 min	1.00	0.00	
		10 min	0.88	0.10	***p = 0.00021
		20 min	0.79	0.17	**p = 0.0013
		30 min	0.71	0.10	***p = 0.00091
	WT	[§]0 min	1.00	0.00	
		10 min	0.04	0.06	
		20 min	0.01	0.02	
		30 min	0.11	0.06	

[§]set to 1

T-test, unpaired, two-tailed

[†]stimulation times were included into the quantitative analysis, which were tested in all biological replicates

	Antibody	Sample	IL-1	Mean (n=3)	±SD	p-value (*IKK2* -/- vs. corresponding WT)
**Figure 2d**	P-JNK	*IKK2* -/-	0 min	0.00	0.00	
			5 min	0.28	0.22	p = 0.63
			10 min	0.86	0.25	p = 0.52
			15 min	1.10	0.29	p = 0.71
			20 min	0.74	0.55	p = 0.46
			30 min	0.39	0.19	p = 0.81
			45 min	0.10	0.05	p = 0.74
			60 min	0.11	0.10	p = 0.76
		WT	0 min	0.00	0.00	
			5 min	0.45	0.50	
			10 min	0.99	0.19	
			15 min	0.99	0.40	
			[§]20 min	1.00	0.00	
			30 min	0.45	0.37	
			45 min	0.12	0.11	
			60 min	0.14	0.13	

[§]set to 1

T-test, unpaired, two-tailed

Antibody	Sample	IL-1	Mean (n=3)	±SD	p-value (*IKK2* -/- vs. corresponding WT)
IκBα	*IKK2* -/-	[§]0 min	1.00	0.00	
		5 min	0.62	0.11	p = 0.084
		10 min	0.42	0.05	***p = 0.00014
		15 min	0.24	0.08	**p = 0.0071
		20 min	0.15	0.09	p = 0.054
		30 min	0.14	0.08	p = 0.21
		45 min	0.68	0.35	p = 0.61
		60 min	0.89	0.23	p = 0.46
	WT	[§]0 min	1.00	0.00	
		5 min	0.35	0.18	
		10 min	0.00	0.01	
		15 min	0.00	0.01	
		20 min	0.01	0.01	
		30 min	0.06	0.06	
		45 min	0.55	0.24	
		60 min	1.08	0.32	

[§]set to 1

T-test, unpaired, two-tailed

	Antibody	Sample	X-link	Mean (n=2)	±SD	p-value (vs. corresponding WT)
**Figure 2e**	P-JNK	WT	0 min	0.04	0.01	
			30 min	0.11	0.13	
			60 min	0.55	0.28	
			90 min	0.87	0.12	
			120 min	0.97	0.15	
			§180 min	1.00	0.00	
		IKK1 crKO	0 min	0.00	0.00	
			30 min	0.03	0.04	p = 0.47
			60 min	0.42	0.16	p = 0.61
			90 min	0.75	0.14	p = 0.45
			120 min	0.44	0.14	p = 0.068
			180 min	0.46	0.17	*p = 0.043
		IKK2 -/-	0 min	0.00	0.00	
			30 min	0.00	0.00	p = 0.35
			60 min	0.00	0.00	p = 0.11
			90 min	0.17	0.15	*p = 0.035
			120 min	0.19	0.16	*p = 0.037
			180 min	0.09	0.06	**p = 0.0019
		IKK1 crKO/*IKK2* -/-	0 min	0.00	0.00	
			30 min	0.00	0.00	p = 0.35
			60 min	0.01	0.01	p = 0.11
			90 min	0.19	0.16	*p = 0.041
			120 min	0.00	0.00	*p = 0.012
			180 min	0.03	0.04	***p = 0.00094

§set to 1 — T-test, unpaired, two-tailed

Antibody	Sample	X-link	Mean (n=2)	±SD	p-value (vs. corresponding WT)
IκBα	WT	§0 min	1.00	0.00	
		30 min	0.89	0.27	
		60 min	0.27	0.11	
		90 min	0.13	0.16	
		120 min	0.24	0.17	
		180 min	0.32	0.00	
	IKK1 crKO	§0 min	1.00	0.00	
		30 min	0.15	0.16	p = 0.08
		60 min	0.07	0.10	p = 0.19
		90 min	0.14	0.05	p = 0.94
		120 min	0.21	0.08	p = 0.81
		180 min	0.26	0.15	p = 0.68
	IKK2 -/-	§0 min	1.00	0.00	
		30 min	0.84	0.01	p = 0.81
		60 min	0.89	0.06	*p = 0.019
		90 min	0.80	0.10	*p = 0.036
		120 min	0.81	0.01	*p = 0.043
		180 min	0.88	0.09	*p = 0.011
	IKK1 crKO/*IKK2* -/-	§0 min	1.00	0.00	
		30 min	1.95	0.34	p = 0.073
		60 min	1.84	0.21	*p = 0.01
		90 min	2.06	0.73	p = 0.067
		120 min	1.22	0.59	p = 0.15
		180 min	0.97	0.47	p = 0.18

§set to 1 — T-test, unpaired, two-tailed

	Antibody	Sample	X-link	Mean (n=2 WT, n=5 *NEMO* ^{crKO} clones)	±SD	p-value (*NEMO* ^{crKO} vs. corresponding WT)
**Figure 2f**	P-JNK	WT	0 min	0.01	0.01	
			30 min	0.15	0.22	
			60 min	0.48	0.35	
			90 min	0.84	0.40	
			§120 min	1.00	0.00	
			180 min	0.61	0.21	
		NEMO ^{crKO}	0 min	0.00	0.00	
			30 min	0.00	0.00	p = 0.12
			60 min	0.00	0.00	*p = 0.015
			90 min	0.00	0.00	**p = 0.0024
			120 min	0.04	0.08	****p = 0.000015
			180 min	0.08	0.16	*p = 0.013

§set to 1

T-test, unpaired, two-tailed

Antibody	Sample	X-link	Mean (n=2 WT, n=5 *NEMO* ^{crKO} clones)	±SD	p-value (*NEMO* ^{crKO} vs. corresponding WT)
IκBα	WT	§0 min	1.00	0.00	
		30 min	0.65	0.33	
		60 min	0.32	0.41	
		90 min	0.14	0.03	
		120 min	0.44	0.37	
		180 min	0.10	0.11	
	NEMO ^{crKO}	§0 min	1.00	0.00	
		30 min	0.76	0.16	p = 0.56
		60 min	0.70	0.43	p = 0.34
		90 min	0.54	0.18	*p = 0.03
		120 min	0.54	0.22	p = 0.66
		180 min	0.81	0.57	p = 0.16

§set to 1

T-test, unpaired, two-tailed

	Antibody	Sample	X-link	Mean (n=3)	±SD	p-value (TAK1-IH vs. corresponding DMSO)
**Figure 3a**	P-JNK	DMSO	0 min	0.00	0.00	
			30 min	0.08	0.13	
			60 min	0.43	0.68	
			90 min	0.86	0.61	
			§120 min	1.00	0.00	
		TAK1-IH	0 min	0.00	0.00	
			30 min	0.03	0.04	p = 0.56
			60 min	0.00	0.00	p = 0.33
			90 min	0.00	0.01	p = 0.073
			120 min	0.04	0.06	****p = 0.000011

§set to 1

T-test, unpaired, two-tailed

Antibody	Sample	X-link	Mean (n=3)	±SD	p-value (TAK1-IH vs. corresponding DMSO)
IκBα	DMSO	§0 min	1.00	0.00	
		30 min	0.61	0.20	
		60 min	0.36	0.27	
		90 min	0.21	0.08	
		120 min	0.20	0.05	
	TAK1-IH	§0 min	1.00	0.00	
		30 min	0.64	0.16	p = 0.85
		60 min	0.26	0.11	p = 0.59
		90 min	0.16	0.11	p = 0.55
		120 min	0.07	0.07	p = 0.077

§set to 1

T-test, unpaired, two-tailed

	Antibody	Sample	X-link	Mean (n=3)	±SD	p-value (vs. LMP1 wt, WT)
**Figure 3b**	P-IKK2 S177/181	WT + TAK1-IH	vector	0.00	0.00	
			LMP1 wt	0.86	0.36	p = 0.57
			AAA/Y_{384}G	0.15	0.24	
		WT	vector	0.00	0.00	
			§LMP1 wt	1.00	0.00	
			AAA/Y_{384}G	0.09	0.08	
		TAK1 ^{crKO}	vector	0.00	0.00	
			LMP1 wt	0.00	0.00	no p-value determinable vs. WT
			AAA/Y_{384}G	0.00	0.00	

§set to 1

T-test, unpaired, two-tailed

	Antibody	Sample		Mean (n=4 DMSO, ACHP, n=3 TAK1-IH)	±SD	p-value (vs. LMP1 wt, DMSO)
**Figure 3d**	P-IKK2 S177/181	DMSO	§LMP1 wt	1	0	
			vector	0.19	0.23	
		ACHP	LMP1 wt	0.13	0.22	***p = 0.0002
			vector	0.00	0.00	
		TAK1-IH	LMP1 wt	1.18	0.49	p = 0.50
			vector	0.28	0.37	
			§set to 1			T-test, unpaired, two-tailed

	Antibody	Sample		Mean (n=2)	±SD	p-value (vs. LMP1 wt, WT)
**Figure 3e**	Ubiquitin	WT + TAK1-IH	w/o	0.09	0.07	
			LMP1 wt	0.74	0.11	p = 0.083
		WT	w/o	0.19	0.27	
			§LMP1 wt	1.00	0.00	
		TAK1 ^{crKO}	w/o	0.03	0.04	
			LMP1 wt	0.07	0.09	**p = 0.0049
			§set to 1			T-test, unpaired, two-tailed

**Figure 3f**	Antibody	Sample		Mean (n=3)	±SD	p-value (vs. LMP1wt WT)
	LMP1	WT	§LMP1 wt	1.00	0.00	
		WT	LMP1 AAA/Δ371	0.03	0.04	
		TAK1 ^{crKO}	LMP1wt	0.03	0.04	**** p < 0.0001
		TAK1 ^{crKO}	LMP1 AAA/Δ371	0.00	0.00	
				§set to 1		T-test, unpaired, two-tailed

**Figure 4a**	Antibody		Sample	Mean (n=4)	±SD	p-value (vs. LMP1 WT)
	P-TPL2 S400		§LMP1 WT	1	0	
			LMP1 AAA/Y_{384}G	0.48	0.36	*p = 0.029
			§set to 1			T-test, unpaired, two-tailed

**Figure 4b**	Antibody	X-link	n=1
	P-TPL2 S400	0 min	0
		§30 min	1
		60 min	0
		§set to 1	

	Antibody	Sample	X-link	Mean (n=5, n=2 for 3 h)	±SD	p-value (vs. 0 h)
**Figure 4c**	TPL2	NGFR-LMP1wt	§0 h	1	0	
			1 h	0.36	0.23	**p = 0.0032
			2 h	0.10	0.07	****p < 0.00001
			3 h	0.06	0.07	*p = 0.032
			§set to 1			T-test, unpaired, two-tailed

	Antibody	Sample	X-Link	Mean (n=2[†])	±SD	p-value (60 min vs. 0 min)
**Figure 4d**	TPL2	TPL2 coIP	[§]0 min	1	0	
		TPL2 coIP	60 min	0.42	0.26	p = 0.086
			[§]set to 1			T-test, unpaired, two-tailed

[†]stimulation times were included into the quantitative analysis, which were tested in both biological replicates

	Antibody	Sample	X-Link	Mean (n=2)	±SD	p-value (siIKK2 vs. corresponding siCTRL)	
**Figure 4e**	TPL2	siCTRL	[§]0 h	1.00	0.00		
			1 h	0.31	0.25		
			2 h	0.26	0.24		
		siIKK2	[§]0 h	1.00	0.00		
			1 h	1.09	0.11	p = 0.055	**p = 0.0072
			2 h	0.93	0.11	p = 0.072	*p = 0.015
			[§]set to 1			T-test, unpaired, two-tailed	two-way ANOVA

	Antibody	Sample	X-Link	Mean (n=2)	±SD	p-value (ACHP vs. corresponding DMSO)
**Figure 4f**	TPL2	DMSO	[§]0 h	1.00	0.00	
			1 h	0.32	0.34	
			2 h	0.10	0.12	
		ACHP	[§]0 h	1.00	0.00	
			1 h	1.04	0.20	p = 0.12
			2 h	0.88	0.20	*p = 0.041
			[§]set to 1			T-test, unpaired, two-tailed

	Antibody	Sample	X-Link	Mean (n=2)	±SD	p-value (*IKK2* -/- vs. corresponding WT)
**Figure 4g**	TPL2	WT	[§]0 h	1.00	0.00	
			1 h	0.50	0.24	
			2 h	0.14	0.15	
			3 h	0.08	0.09	
		IKK2 -/-	[§]0 h	1.00	0.00	
			1 h	1.01	0.10	p = 0.1
			2 h	0.93	0.14	*p = 0.033
			3 h	1.33	0.06	**p = 0.0037
			[§]set to 1			T-test, unpaired, two-tailed

	Antibody	Sample	X-Link	Mean (n=3[†])	±SD	p-value (siTPL2 vs. corresponding siCTRL)
**Figure 5a**	P-JNK	siCTRL	0 min	0.00	0.00	
			30 min	0.00	0.00	
			60 min	0.04	0.04	
			[§]120 min	1.00	0.00	
		siTPL2	0 min	0.00	0.00	
			30 min	0.00	0.00	
			60 min	0.04	0.05	p = 0.91
			120 min	0.42	0.12	**p = 0.0011

[§]set to 1

T-test, unpaired, two-tailed

[†]stimulation times were included into the quantitative analysis, which were tested in all biological replicates

Antibody	Sample	X-Link	Mean (n=3[†])	±SD	p-value (siCTRL vs. corresponding siTPL2)
IκBα	siCTRL	[§]0 min	1.00	0.00	
		30 min	0.74	0.34	
		60 min	0.29	0.23	
		120 min	0.20	0.12	
	siTPL2	[§]0 min	1.00	0.00	
		30 min	0.75	0.20	p = 0.96
		60 min	0.31	0.24	p = 0.92
		120 min	0.24	0.20	p = 0.77

[§]set to 1

T-test, unpaired, two-tailed

[†]stimulation times were included into the quantitative analysis, which were tested in all biological replicates

	Antibody	Sample	X-Link	Mean (n=4)	±SD	p-value (TPL2-IH vs. corresponding DMSO)
**Figure 5b**	P-JNK	DMSO	0 min	0.02	0.04	
			30 min	0.03	0.04	
			60 min	0.11	0.06	
			90 min	0.42	0.09	
			[§]120 min	1.00	0.00	
			180 min	1.14	0.12	
		TPL2-IH	0 min	0.00	0.00	
			30 min	0.00	0.00	p = 0.16
			60 min	0.09	0.05	p = 0.6
			90 min	0.21	0.11	*p = 0.025
			120 min	0.35	0.10	****p = 0.000012
			180 min	0.20	0.08	****p = 0.00001

[§]set to 1

T-test, unpaired, two-tailed

Antibody	Sample	X-Link	Mean (n=4)	±SD	p-value (TPL2-IH vs. corresponding DMSO)
IκBα	DMSO	[§]0 min	1.00	1.00	
		30 min	0.50	0.50	
		60 min	0.18	0.18	
		90 min	0.22	0.22	
		120 min	0.25	0.25	
		180 min	0.29	0.29	
	TPL2-IH	[§]0 min	1.00	0.00	
		30 min	0.64	0.21	p = 0.23
		60 min	0.43	0.23	p = 0.07
		90 min	0.28	0.22	p = 0.59
		120 min	0.36	0.31	p = 0.55
		180 min	0.29	0.19	p = 0.99

[§]set to 1

T-test, unpaired, two-tailed

	Antibody	Sample	X-Link	Mean (n=2[†])	±SD	p-value (*TPL2* crKO vs. corresponding WT)
**Figure 5c**	P-JNK	*TPL2* crKO	0 min	0.00	0.00	
			30 min	0.01	0.01	*p = 0.020
			60 min	0.10	0.14	p = 0.15
			90 min	0.03	0.01	****p = 0.000032
		WT	0 min	0.00	0.01	
			30 min	0.10	0.02	
			60 min	0.41	0.14	
			[§]90 min	1.00	0.00	

[§]set to 1

T-test, unpaired, two-tailed

[†]stimulation times were included into the quantitative analysis, which were tested in both biological replicates

Antibody	Sample	X-Link	Mean (n=2[†])	±SD	p-value (*TPL2* crKO vs. corresponding WT)
IκBα	*TPL2* crKO	[§]0 min	1.00	0.00	
		60 min	0.27	0.06	p = 0.064
		120 min	0.28	0.10	p = 0.66
	WT	[§]0 min	1.00	0.00	
		60 min	0.47	0.05	
		120 min	0.48	0.56	

[§]set to 1

T-test, unpaired, two-tailed

[†]stimulation times were included into the quantitative analysis, which were tested in both biological replicates

	Antibody	Sample		Mean (n=3)	±SD	p-value (*TPL2* crKO vs. WT)
**Figure 5d**	p65	WT, nucleus	[§]LMP1 WT	1.00	0.00	
		TPL2 crKO, nucleus	LMP1 WT	1.72	0.32	p = 0.059
	p50	WT, nucleus	[§]LMP1 WT	1.00	0.00	
		TPL2 crKO, nucleus	LMP1 WT	0.77	0.25	p = 0.18
	RelB	WT, nucleus	[§]LMP1 WT	1.00	0.00	
		TPL2 crKO, nucleus	LMP1 WT	1.18	1.19	p = 0.80
	p52	WT, nucleus	[§]LMP1 WT	1.00	0.00	
		TPL2 crKO, nucleus	LMP1 WT	0.88	0.83	p = 0.81

[§]set to 1

T-test, unpaired, two-tailed

	Antibody	Sample	X-link	Mean (n=2[†])	±SD	p-value (ACHP vs. corresponding DMSO)
**Figure 6a**	P-JNK	DMSO	0 min	0.06	0.08	
			[§]30 min	1.00	0.00	
			60 min	0.27	0.38	
			90 min	0.06	0.09	
			120 min	0.18	0.25	
		ACHP	0 min	0.02	0.03	
			30 min	0.19	0.24	*p = 0.041
			60 min	0.15	0.22	p = 0.75
			90 min	0.07	0.11	p = 0.92
			120 min	0.06	0.08	p= 0.59

[§]set to 1

T-test, unpaired, two-tailed

[†]stimulation times were included into the quantitative analysis, which were tested in all biological replicates

Antibody	Sample	X-link	Mean (n=2[†])	±SD	p-value (ACHP vs. corresponding DMSO)
IκBα	DMSO	[§]0 min	1.00	0.00	
		30 min	0.41	0.15	
		60 min	0.35	0.00	
		90 min	0.57	0.05	
		120 min	0.49	0.28	
	ACHP	[§]0 min	1.00	0.00	
		30 min	1.19	0.40	p = 0.12
		60 min	1.63	0.67	p = 0.11
		90 min	1.37	0.01	**p = 0.002
		120 min	1.71	0.03	*p = 0.026

[§]set to 1

T-test, unpaired, two-tailed

[†]stimulation times were included into the quantitative analysis, which were tested in all biological replicates

Antibody	Sample	X-link	Mean (n=3[†])	±SD	p-value (DMSO vs. corresponding ACHP)
P-ERK	DMSO	0 min	0.11	0.13	
		[§]30 min	1.00	0.00	
		60 min	0.56	0.52	
		90 min	0.48	0.67	
		120 min	0.61	0.77	
	ACHP	0 min	0.44	0.54	
		30 min	0.38	0.33	*p = 0.032
		60 min	0.39	0.40	p = 0.67
		90 min	0.29	0.43	p = 0.71
		120 min	0.13	0.18	p= 0.35

[§]set to 1

T-test, unpaired, two-tailed

[†]stimulation times were included into the quantitative analysis, which were tested in all biological replicates

	Antibody	Sample	CD40L	Mean (n=2)	±SD	p-value (ACHP vs. corresponding DMSO)
**Figure 6b**	P-JNK	DMSO	0 min	0.00	0.00	
			5 min	0.24	0.14	
			§15 min	1.00	0.00	
			30 min	0.33	0.47	
			45 min	0.02	0.02	
		ACHP	0 min	0.00	0.00	
			5 min	0.29	0.22	p = 0.8
			15 min	0.99	0.54	p = 0.98
			30 min	0.35	0.49	p = 0.98
			45 min	0.20	0.29	p = 0.46

§set to 1 T-test, unpaired, two-tailed

Antibody	Sample	CD40L	Mean (n=2)	±SD	p-value (ACHP vs. corresponding DMSO)
IκBα	DMSO	§0 min	1.00	0.00	
		5 min	0.55	0.26	
		15 min	0.22	0.23	
		30 min	0.03	0.02	
		45 min	0.60	0.11	
	ACHP	§0 min	1.00	0.00	
		5 min	0.85	0.03	p = 0.24
		15 min	0.82	0.19	p = 0.10
		30 min	0.86	0.06	**p = 0.0025
		45 min	0.89	0.01	p = 0.067

§set to 1 T-test, unpaired, two-tailed

Antibody	Sample	CD40L	Mean (n=2)	±SD	p-value (ACHP vs. DMSO)
P-ERK	DMSO	0 min	0.02	0.02	
		5 min	0.14	0.20	
		§15 min	1.00	0.00	
		30 min	2.68	3.44	
		45 min	0.06	0.08	
	ACHP	0 min	0.07	0.09	
		5 min	0.07	0.10	p = 0.69
		15 min	0.18	0.25	*p = 0.044
		30 min	0.08	0.11	p = 0.40
		45 min	0.16	0.22	p= 0.61

§set to 1 T-test, unpaired, two-tailed

	Antibody	Sample	X-link	Mean (n=3)	±SD	p-value (TPL2-IH vs. corresponding DMSO)
**Figure 6c**	P-JNK	DMSO	0 min	0.29	0.35	
			§30 min	1.00	0.00	
			60 min	1.06	0.93	
			90 min	0.37	0.30	
			120 min	0.19	0.21	
		TPL2-IH	0 min	0.10	0.09	
			30 min	0.23	0.18	**p = 0.0016
			60 min	0.24	0.31	p = 0.22
			90 min	0.08	0.09	p = 0.18
			120 min	0.03	0.04	p = 0.25
			§set to 1			T-test, unpaired, two-tailed

Antibody	Sample	X-link	Mean (n=3)	±SD	p-value (TPL2-IH vs. corresponding DMSO)
IκBα	DMSO	§0 min	1.00	0.00	
		30 min	0.76	0.38	
		60 min	0.34	0.15	
		90 min	0.32	0.07	
		120 min	0.67	0.09	
	TPL2-IH	§0 min	1.00	0.00	
		30 min	0.83	0.30	p = 0.82
		60 min	0.32	0.10	p = 0.84
		90 min	0.31	0.14	p = 0.88
		120 min	0.57	0.35	p = 0.63
		§set to 1			T-test, unpaired, two-tailed

Antibody	Sample	X-link	Mean (n=3)	±SD	p-value (TPL2-IH vs. corresponding DMSO)
P-ERK	DMSO	0 min	0.44	0.68	
		§30 min	1.00	0.00	
		60 min	1.12	1.67	
		90 min	0.31	0.45	
		120 min	0.16	0.19	
	TPL2-IH	0 min	0.18	0.30	p = 0.58
		30 min	0.28	0.26	**p = 0.0085
		60 min	0.85	1.26	p = 0.83
		90 min	0.19	0.27	p = 0.73
		120 min	0.20	0.29	p = 0.87
		§set to 1			T-test, unpaired, two-tailed

	Antibody	Sample	CD40L	Mean (n=2†)	±SD	p-value (TPL2-IH vs. corresponding DMSO)
**Figure 6d**	P-JNK	DMSO	0 min	0.00	0.00	
			5 min	0.41	0.29	
			§15 min	1.00	0.00	
			30 min	0.84	0.21	
		TPL2-IH	0 min	0.01	0.01	
			5 min	0.34	0.31	p = 0.82
			15 min	0.85	0.18	p = 0.36
			30 min	0.59	0.51	p = 0.59

§set to 1

T-test, unpaired, two-tailed

†stimulation times were included into the quantitative analysis, which were tested in both biological replicates

Antibody	Sample	CD40L	Mean (n=2†)	±SD	p-value (TPL2-IH vs. corresponding DMSO)
IκBα	DMSO	§0 min	1.00	0.00	
		5 min	0.38	0.42	
		15 min	0.02	0.03	
		30 min	0.12	0.16	
	TPL2-IH	§0 min	1.00	0.00	
		5 min	0.45	0.62	p = 0.91
		15 min	0.03	0.05	p = 0.82
		30 min	0.00	0.00	p = 0.40

§set to 1

T-test, unpaired, two-tailed

†stimulation times were included into the quantitative analysis, which were tested in both biological replicates

Antibody	Sample	CD40L	Mean (n=2†)	±SD	p-value (TPL2-IH vs. corresponding DMSO)
P-ERK	DMSO	0 min	0.00	0.00	
		5 min	0.21	0.30	
		§15 min	1.00	0.00	
		30 min	0.39	0.51	
	TPL2-IH	0 min	0.00	0.00	
		5 min	0.00	0.00	p = 0.42
		15 min	0.14	0.20	*p = 0.026
		30 min	0.15	0.21	p = 0.60

§set to 1

T-test, unpaired, two-tailed

†stimulation times were included into the quantitative analysis, which were tested in both biological replicates

	Antibody	Sample	X-link	Mean (n=3 DMSO, n=5 TPL2-IH)	±SD	p-value (TPL2-IH vs. corresponding DMSO)
**Figure 6f**	P-JNK	DMSO	0 min	0.00	0.01	
			30 min	0.16	0.15	
			§60 min	1.00	0.00	
			90 min	0.40	0.07	
		TPL2-IH	0 min	0.01	0.01	
			30 min	0.05	0.02	p = 0.14
			60 min	0.34	0.18	***p = 0.00076
			90 min	0.17	0.12	*p = 0.024
			§set to 1			T-test, unpaired, two-tailed

Antibody	Sample	X-link	Mean (n=2)	±SD	p-value (TPL2-IH vs. corresponding DMSO)
IκBα	DMSO	§0 min	1.00	0.00	
		30 min	0.72	0.10	
		60 min	0.44	0.08	
		90 min	0.50	0.13	
	TPL2-IH	§0 min	1.00	0.00	
		30 min	0.72	0.20	p = 0.99
		60 min	0.43	0.09	p = 0.87
		90 min	0.29	0.05	p = 0.17
		§set to 1			T-test, unpaired, two-tailed

Antibody	Sample	X-link	Mean (n=2)	±SD	p-value (ACHP vs. corresponding DMSO)
P-JNK	DMSO	0 min	0.01	0.02	
		30 min	0.18	0.12	
		§60 min	1.00	0.00	
		90 min	0.55	0.32	
	ACHP	0 min	0.02	0.03	
		30 min	0.02	0.03	p = 0.20
		60 min	0.03	0.05	**p = 0.0011
		90 min	0.02	0.02	p = 0.14
		§set to 1			T-test, unpaired, two-tailed

Antibody	Sample	X-link	Mean (n=3)	±SD	p-value (ACHP vs. corresponding DMSO)
IκBα	DMSO	§0 min	1.00	0.00	
		30 min	0.44	0.37	
		60 min	0.40	0.08	
		90 min	0.44	0.22	
	ACHP	§0 min	1.00	0.00	
		30 min	0.93	0.01	p = 0.088
		60 min	0.89	0.11	**p = 0.004
		90 min	0.98	0.28	p = 0.058
		§set to 1			T-test, unpaired, two-tailed

Supplementary Table 4. Phosphoimager quantification and statistics of kinase activities (Figures 3b and 3c)

n, number of biological replicates
SD, standard deviation

	Substrate phosphorylation	Sample		Mean (n=3)	±SD	p-value
**Figure 3b**	IKK2 kinase activity, GST-IκBα~P*	TAK1-IH	vector	0.14	0.06	
			LMP1 wt	2.59	0.89	p = 0.75 (TAK1-IH vs. WT); **p = 0.008 (TAK1-IH vs. *TAK1* crKO)
			AAA/Y_{384}G	0.79	0.13	
		WT	vector	0.14	0.06	
			LMP1 wt	2.93	1.46	
			§AAA/Y_{384}G	1.00	0.00	
		TAK1 crKO	vector	0.07	0.03	
			LMP1 wt	0.06	0.04	*p = 0.027 (*TAK1* crKO vs. WT)
			AAA/Y_{384}G	0.07	0.03	
			§set to 1			T-test, unpaired, two-tailed

	Substrate phosphorylation	Sample		Mean (n=3)	±SD	p-value
**Figure 3c**	JNK1 kinase activity, GST-c-Jun~P*	TAK1-IH	vector	0.49	0.13	
			LMP1 wt	0.78	0.73	*p = 0.037 (TAK1-IH vs. WT); p = 0.64 (TAK1-IH vs. *TAK1* crKO)
			AAA/Y_{384}G	0.47	0.15	
		WT	vector	0.72	0.20	
			LMP1 wt	11.28	5.84	
			§AAA/Y_{384}G	1.00	0.00	
		TAK1 crKO	vector	0.81	0.50	
			LMP1 wt	1.10	0.84	*p = 0.041 (*TAK1* crKO vs. WT)
			AAA/Y_{384}G	1.23	0.71	
			§set to 1			T-test, unpaired, two-tailed

Supplementary References

1. Sandvej, K. *et al.* Sequence analysis of the Epstein-Barr virus (EBV) latent membrane protein-1 gene and promoter region: identification of four variants among wild-type EBV isolates. *Blood* **90**, 323-330 (1997).
2. Mainou, B. A. & Raab-Traub, N. LMP1 strain variants: biological and molecular properties. *J Virol* **80**, 6458-6468 (2006).
3. Guiretti, D. M. *et al.* Structural variability of the carboxy-terminus of Epstein-Barr virus encoded latent membrane protein 1 gene in Hodgkin's lymphomas. *J Med Virol* **79**, 1730-1722 (2007).
