# Supplementary material for: A central role of IKK2 and TPL2 in JNK activation and viral B-cell transformation
Source: Nat Commun. 2020 Feb 4;11:685. doi: 10.1038/s41467-020-14502-x (PMC7000802; doi:10.1038/s41467-020-14502-x)
Supplement: Supplementary file 2 — Reporting Summary [file 41467_2020_14502_MOESM2_ESM.pdf]

nature research

Corresponding author(s): Arnd Kieser
Last updated by author(s): Dec 3, 2019

Reporting Summary

Nature Research wishes to improve the reproducibility of the work that we publish. This form provides structure for consistency and transparency in reporting. For further information on Nature Research policies, see Authors & Referees and the Editorial Policy Checklist.

Statistics

For all statistical analyses, confirm that the following items are present in the figure legend, table legend, main text, or Methods section.

n/a	Confirmed	
☐	☒	The exact sample size (*n*) for each experimental group/condition, given as a discrete number and unit of measurement
☐	☒	A statement on whether measurements were taken from distinct samples or whether the same sample was measured repeatedly
☐	☒	The statistical test(s) used AND whether they are one- or two-sided<br>*Only common tests should be described solely by name; describe more complex techniques in the Methods section.*
☒	☐	A description of all covariates tested
☒	☐	A description of any assumptions or corrections, such as tests of normality and adjustment for multiple comparisons
☐	☒	A full description of the statistical parameters including central tendency (e.g. means) or other basic estimates (e.g. regression coefficient) AND variation (e.g. standard deviation) or associated estimates of uncertainty (e.g. confidence intervals)
☒	☐	For null hypothesis testing, the test statistic (e.g. *F*, *t*, *r*) with confidence intervals, effect sizes, degrees of freedom and *P* value noted<br>*Give P values as exact values whenever suitable.*
☒	☐	For Bayesian analysis, information on the choice of priors and Markov chain Monte Carlo settings
☒	☐	For hierarchical and complex designs, identification of the appropriate level for tests and full reporting of outcomes
☒	☐	Estimates of effect sizes (e.g. Cohen's *d*, Pearson's *r*), indicating how they were calculated

Our web collection on statistics for biologists contains articles on many of the points above.

Software and code

Policy information about availability of computer code

Data collection	Flow cytometry: BD FACSDiva 8.0.1 (BD FACSCanto II), CellQuest Pro 6.0 (BD FACS Calibur). Software used for imager-based immunoblot capture: Vilber EvolutionCapt Edge 18.02
Data analysis	Flow Cytometry: FlowJo X.0.7, imager-based immunoblot quantification: Vilber Bio-1D 15.08, immunoblot densitometry: ImageJ 1.51, further data analysis and statistics: Microsoft Excel and GraphPad Prism 6.0

For manuscripts utilizing custom algorithms or software that are central to the research but not yet described in published literature, software must be made available to editors/reviewers. We strongly encourage code deposition in a community repository (e.g. GitHub). See the Nature Research guidelines for submitting code & software for further information.

Data

Policy information about availability of data

All manuscripts must include a data availability statement. This statement should provide the following information, where applicable:

- Accession codes, unique identifiers, or web links for publicly available datasets
- A list of figures that have associated raw data
- A description of any restrictions on data availability

LMP1 sequencing data have been deposited in GenBank under the accession codes MN244676 for PTLD099 and MN244677 for PTLD880. Original uncropped scans of all immunoblots and other important original data are supplied in the Source Data file. Original data are included for the following Figures: 1c, 1d, 1e, 1f, 2a, 2b, 2c, 2d, 2e, 2f, 3a, 3b, 3c, 3d, 3e, 3f, 4a, 4b, 4c, 4d, 4e, 4f, 4g, 5a, 5b, 5c, 5d, 6a, 6b, 6c, 6d, 6e, 6f, 7a, 7b, 7c, 7d, 7e, 8a, 8b, 8c, 8d, Supplementary Figures 1a, 1b, 2a, 2b, 3a, 3b, 3c, 4a, 4b, 5a, 5b, and 7.

Field-specific reporting

Please select the one below that is the best fit for your research. If you are not sure, read the appropriate sections before making your selection.

☒ Life sciences ☐ Behavioural & social sciences ☐ Ecological, evolutionary & environmental sciences

For a reference copy of the document with all sections, see nature.com/documents/nr-reporting-summary-flat.pdf

Life sciences study design

All studies must disclose on these points even when the disclosure is negative.

Sample size	No sample size calculation was performed. For cell proliferation, apoptosis, caspase 3 activity assays and imager-based immunoblot analysis (Figures 7a and 8a, 8b) three or more biological replicates (samples) were analysed, which is sufficient to reach statistical significance according to the experience of the authors. For standard immunoblot analysis two or more biological replicates or independent experiments, respectively, were performed and statistically analysed (see Supplementary Table 3).
Data exclusions	No data were excluded.
Replication	All experiments except of Figure 4b, which confirms Figure 4a in the same cell sytem, were performed at least two times or more to guarantee reproducibility of all experiments. The results of all experiments were reproduced.
Randomization	No allocation of samples/patients into experimental groups was performed. No covariates needed to be taken into account.
Blinding	Blinding was not relevant to our study, because allocation of patients/animals into experimental groups was not included in this study.

Reporting for specific materials, systems and methods

We require information from authors about some types of materials, experimental systems and methods used in many studies. Here, indicate whether each material, system or method listed is relevant to your study. If you are not sure if a list item applies to your research, read the appropriate section before selecting a response.

Materials & experimental systems

n/a	Involved in the study	
☐	☒	Antibodies
☐	☒	Eukaryotic cell lines
☒	☐	Palaeontology
☒	☐	Animals and other organisms
☐	☒	Human research participants
☒	☐	Clinical data

Methods

n/a	Involved in the study	
☒	☐	ChIP-seq
☐	☒	Flow cytometry
☒	☐	MRI-based neuroimaging

Antibodies

Antibodies used	The following primary antibodies were used:<br>Abcam: P-JNK1+2+3 Thr183+Thr183+Tyr221 (ab124956, Abcam, used at 1:1000 dilution).<br>BD Pharmingen: Alexa Fluor 647 anti-CD271 (NGFR) (#560877).<br>Cell Signaling Technology: ERK1/2 p44/42 (#9102, 1:1000), P-ERK1/2 Thr202/Tyr204 (#9101, 1:1000), P-JNK Thr183/Tyr185 (#9251, 1:1000), P-JNK Thr183/Tyr185 (#4668, 1:1000), P-c-Jun Ser73 (#3270, 1:1000), P-IKKα/β (IKK1/2) Ser177/181 of IKK2 (#2697, 1:1000), NF-κB p65 (#4764, 1:1000), NF-κB p100/p52 (#4882, 1:1000), P-TPL2 Ser400 (#4491, 1:500), P-p65 Ser536 (#3033, 1:1000).<br>Santa Cruz Biotech.: Cot (TPL2) M20 (sc-720, 1:500), IκBα C21 (sc-371, 1:1000), IKKα (IKK1) M280 (sc-7182, 1:500), IKKα/β (IKK1/2) H470 (sc-7607, 1:500), IKKγ (NEMO) FL419 (sc-8330, 1:500), JNK1/3 C17 (sc-474, 1:500), c-Jun H79 (sc-1694, 1:500), NF-κB p50 C19 (sc-1190, 1:500), RelB C19 (sc-226, 1:500), Sam68 C20 (sc-333, 1:1000), TAK1 M579 (sc-7162, 1:500), TRAF3 C20 (sc-949, 1:500), Tubulin B-5-1-2 (sc-23948, 1:5000).<br>Sigma-Aldrich: HA 3F10 (11867423001, 1:1000), Flag 6F7 (SAB4200071, 1:1000), Myc 9E10 (SAB4700447, 1:1000).<br>Thermo Scientific: NF-κB p105 (PA5-17150, 1:1000).<br>Antibody provided by Elisabeth Kremmer (Monoclonal Antibody Facility of the Helmholtz Centre Munich): rat anti-LMP1 1G6-3 hybridoma supernatant (used at 1:5 dilution).
Validation	Information regarding validation, specificity and references of all commercial antibodies used in this study are available on the websites, in catalogues or datatsheets of the suppliers. The LMP1 antibody 1G6-3 has been used and verified in several published studies, some of which are cited in the paper, e.g. Nicholls, J. et al., J. Virol. Methods 116, 79-88 (2004). All antibodies are approved for the applications and species used in this study.

Eukaryotic cell lines

Policy information about cell lines

Cell line source(s)	HEK293 cells were obtained from the German Collection of Microorganisms and Cell Cultures (DSMZ). IKK2-/- MEFs were provided by Marc Schmidt-Supprian. The lymphoblastoid cell line LCL 1C3 (provided by Josef Mautner) was generated by infection of primary human B cells with B95.8 EBV. BL41:NGFR-LMP1wt cells (provided by Josef Mautner), EBV-negative BL41 Burkitt lymphoma cells and LCL721 have been described and were taken from own laboratory stocks. The LMP1 transgene (LMP1tg)-positive carcinoma cell line 53.234a and corresponding LMP1-negative 53.217 carcinoma cells were established from LMP1tg mice or LMP1-negative siblings, respectively and have been described. References are listed in the paper. PTLD099, PTLD880 and LCL877 cells were established in the course of this study from anonymised tumour biopsies of two adult patients with cytopathologically confirmed EBV-positive PTLD after allogeneic hematopoietic stem cell transplantation. Characterization of these cells is described in the paper.
Authentication	HEK293 cells were directly obtained from the German Collection of Microorganisms and Cell Cultures (DSMZ). All other cells were unique, newly established or not authenticated. Identity of IKK2-/- MEFs and other knockout lines was confirmed by immunoblotting. LCLs were confirmed by immunoblotting for LMP1.
Mycoplasma contamination	Cell lines were not tested for mycoplasma contamination.
Commonly misidentified lines (See ICLAC register)	No commonly misidentified cell lines were used in this study.

Human research participants

Policy information about studies involving human research participants

Population characteristics	The two PTLD patients were adults who had previously received an allogeneic hematopoietic stem cell transplantation and subsequently developed PTLD. No additional selection criteria were applied. No covariate-relevant characteristics were recorded due to sample anonymisation.
Recruitment	Residual material from routine biopsies of two human patients with EBV-positive post-transplant lymphoproliferative disease (PTLD) was anonymised and transferred to our laboratory. Patients had received an allogeneic hematopoietic stem cell transplantation at the Department of Medicine III, University Hospital Grosshadern, Ludwig-Maximilians-University Munich. No additional clinical features or patient-identifying information was recorded. No additional selection criteria were used to recruit patients for this analysis. In addition, peripheral blood from one healthy adult donor was used for generation of NGFR-LMP1 expressing LCLs. The choice of this donor was based upon availability for blood donation and EBV seronegativity. No other selection criteria were applied. Blood collection from healthy donors was likewise approved by the Institutional Review board as specified below.
Ethics oversight	Anonymised human PTLD biopsies and blood from a healthy human donor were obtained with informed consent as approved by the Institutional Review Board (Ethics Commission of the Faculty of Medicine of the Ludwig-Maximilians-University Munich, project no. 071-06 - 075-06).

Note that full information on the approval of the study protocol must also be provided in the manuscript.

Flow Cytometry

Plots

Confirm that:

☒ The axis labels state the marker and fluorochrome used (e.g. CD4-FITC).

☒ The axis scales are clearly visible. Include numbers along axes only for bottom left plot of group (a 'group' is an analysis of identical markers).

☒ All plots are contour plots with outliers or pseudocolor plots.

☒ A numerical value for number of cells or percentage (with statistics) is provided.

Methodology

Sample preparation	B cells were washed in PBS and resuspended in Apoptosis Kit binding buffer and stained using the AnnexinV Apoptosis Kit (Biovision K103) according to the standard protocol provided by the supplier. Adherent carcinoma cells were detached with trypsin, washed with PBS, resuspended in Apoptosis Kit binding buffer. For detection of NGFR-LMP1 at the cell surface of MEFs, cells were mechanically detached from the cell culture plate, washed once with FACS buffer (PBS, 1 % fetal calf serum) and stained with antibody in FACS buffer, or remained unstained as a control.
Instrument	FACSCanto II (Becton Dickinson) for apoptosis assays, FACS Calibur (Becton Dickinson) for NGFR-LMP1 detection in MEFs
Software	Data collection: BD FACSDiva 8.0.1 (BD FACSCanto II), CellQuest Pro 6.0 (BD FACS Calibur).<br>Data analysis: FlowJo X.0.7

Cell population abundance	No sorting steps were performed before analysis by flow cytometry.
Gating strategy	The gating strategy is shown in Supplementary Figure 8. All cells were included into the analysis, whereas cell debris was excluded.

☒ Tick this box to confirm that a figure exemplifying the gating strategy is provided in the Supplementary Information.
